# Supplementary material for: A broadly generalizable stabilization strategy for sarbecovirus fusion machinery vaccines
Source: Nat Commun. 2024 Jun 28;15:5496. doi: 10.1038/s41467-024-49656-5 (PMC11214633; doi:10.1038/s41467-024-49656-5)
Supplement: Supplementary file 1 — Supplementary Information [file 41467_2024_49656_MOESM1_ESM.pdf]

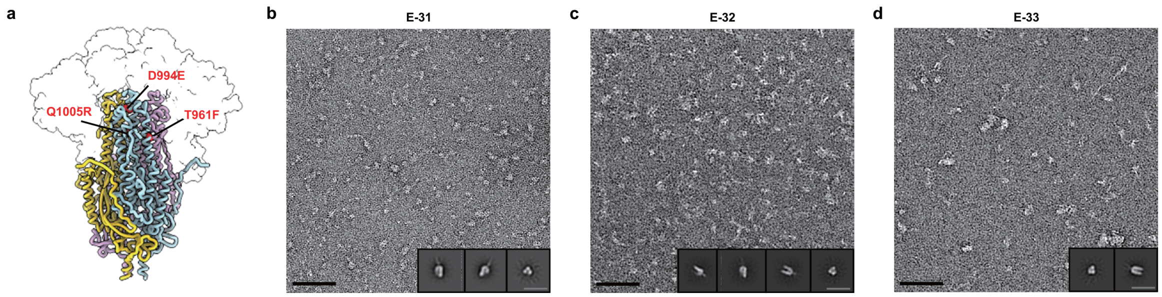

Supplementary Figure 1. Characterization of designed SARS-CoV-2 S_2 prefusion immunogens with single mutations. a, Ribbon diagram of prefusion SARS-CoV-2 S (PDB 6VXX) highlighting all three positions shown in red (T961F, D994E, Q1005R) that were individually mutated to attempt to stabilize the metastable fusion machinery in the prefusion conformation. The S_1 subunit is shown as a semi-transparent surface and glycans are omitted for clarity. **b-d,** EM analysis of negatively stained E-31 (T961F) (b), E-32 (D994E) (c), and E-33 (Q1005R) (d). Insets: 2D class averages showing compact and splayed open prefusion S_2 trimers. The scale bar represents 50 nm (black) or 200 Å (insets, gray).

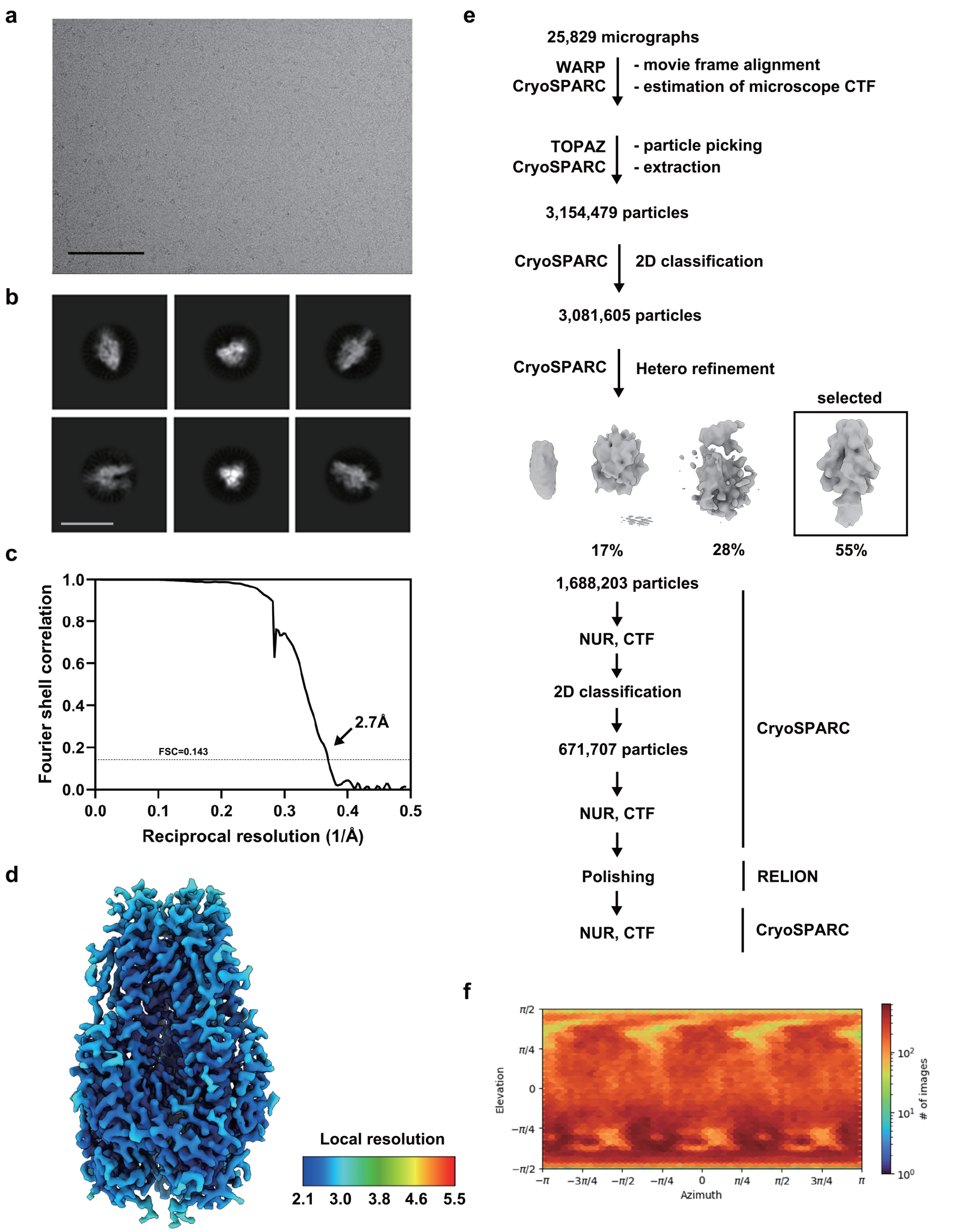

Supplementary Figure 2. CryoEM data collection and refinement of SARS-CoV-2 S_2 E-31. a-b, Representative electron micrograph (a) and 2D class averages (b) of SARS-CoV-2 S_2 E-31 embedded in vitreous ice. The scale bar represents 100 nm (a) or 160Å (b). **c,** Gold-standard Fourier shell correlation curve for the cryoEM reconstruction. The 0.143 cutoff is indicated with a gray dashed line. **d,** SARS-CoV-2 S_2 E-31 cryoEM map colored by local

resolution as determined using cryoSPARC. **e,** Data processing flowchart. NUR, CTF: non-uniform refinement with per-particle defocus refinement. **f,** Angular distribution of E-31 particle images in the final map.

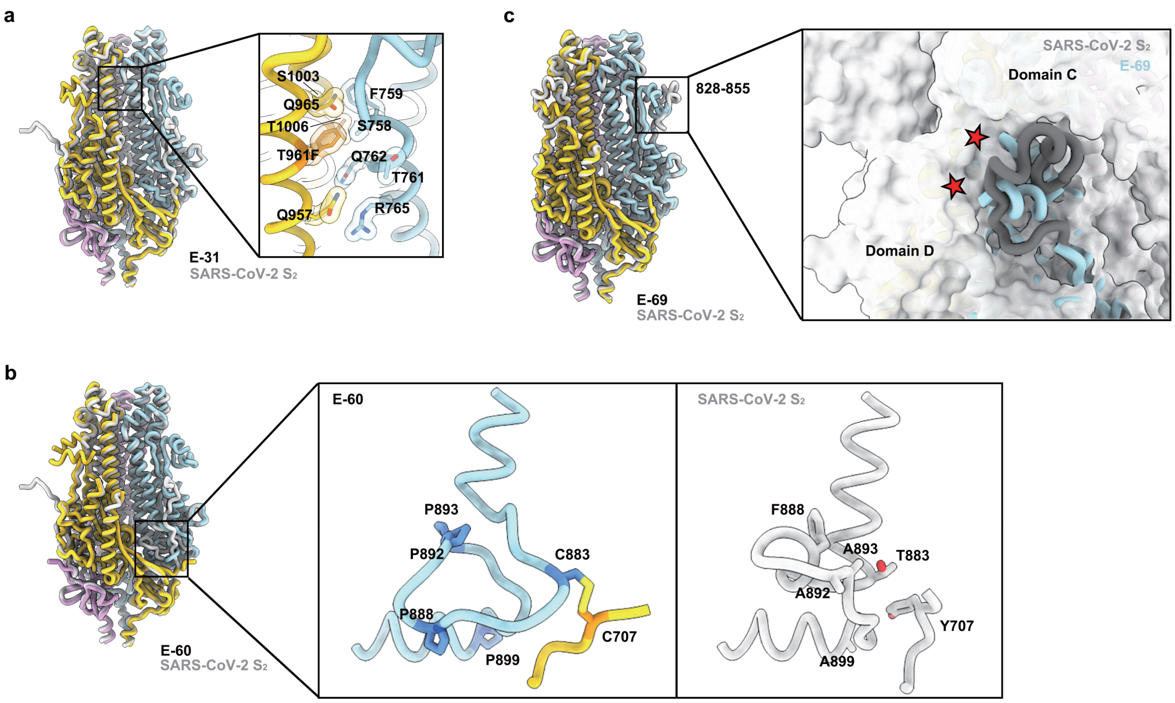

Supplementary Figure 3. Structural details of prefusion-stabilized S_2 subunit designs. **a,** E-31 superimposed to the SARS-CoV-2 S_2 subunit from the S ectodomain trimer (PDB 6VXX, gray). Inset: zoomed-in view of the T961F mutation and proximal residues with selected side chains shown as semi-transparent surfaces. **b,** E-60 superimposed to the SARS-CoV-2 S_2 subunit from the S ectodomain trimer (PDB 6VXX, gray). Inset: zoomed-in view of residues 875-906 of E-60 (left) and SARS-CoV-2 S (right) to highlight local structural distortions. Mutated residues are shown in blue and orange. **c**, E-69 superimposed to the SARS-CoV-2 S_2 subunit from the S ectodomain trimer (PDB 6XR8, residues 705-1146). Steric clashes are indicated with red stars.

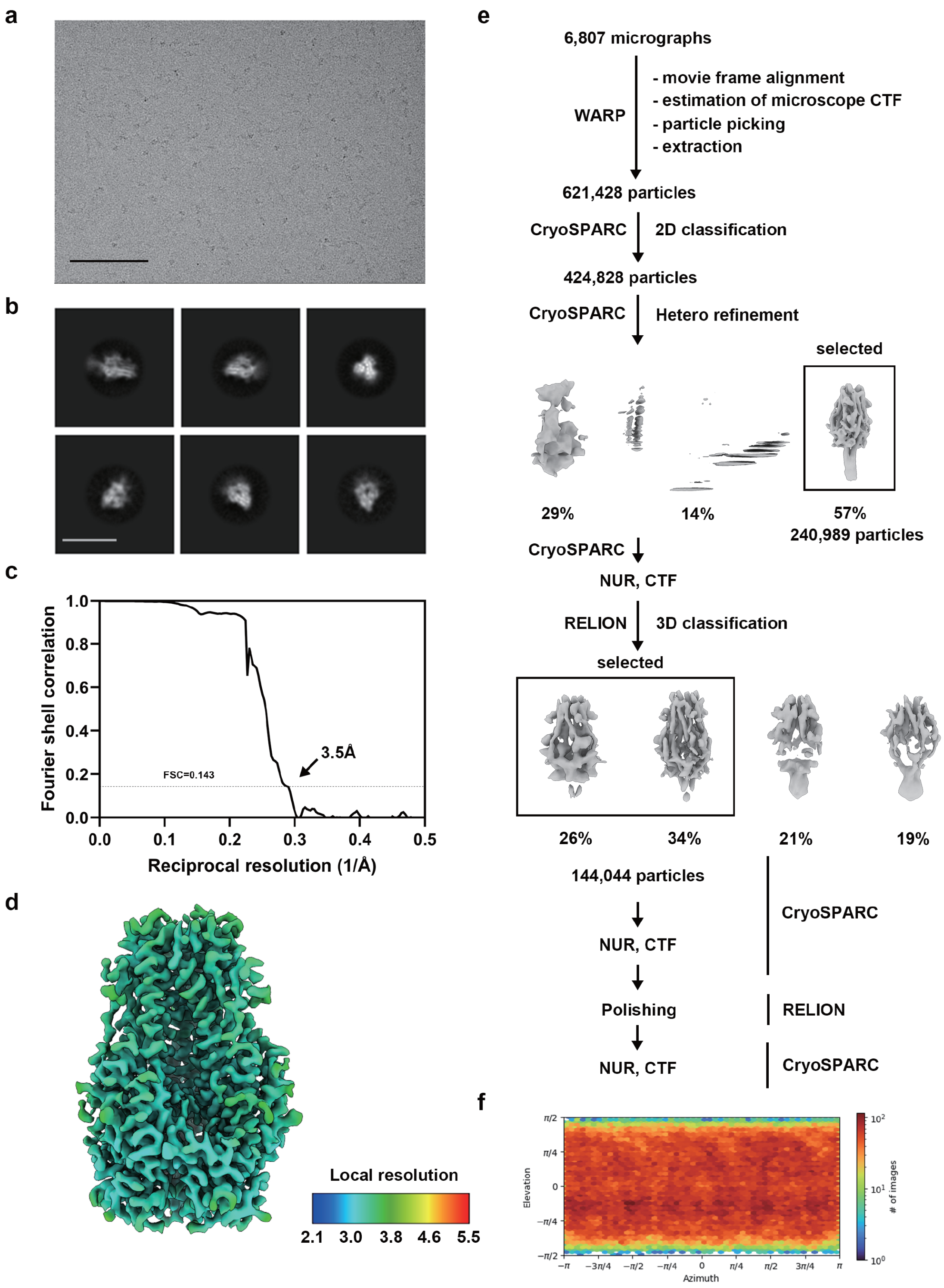

Supplementary Figure 4. CryoEM data collection and refinement of SARS-CoV-2 S_2 E-60. a-b, Representative electron micrograph (a) and 2D class averages (b) of SARS-CoV-2 S_2 E-60 embedded in vitreous ice. The scale bar represents 100 nm (a) or 160Å (b). **c,** Gold-standard Fourier shell correlation curve for the cryoEM reconstruction. The 0.143 cutoff is indicated with a gray dashed line. **d,** SARS-CoV-2 S_2 E-60 cryoEM map colored by local

resolution as determined using cryoSPARC. **e,** Data processing flowchart. NUR, CTF: non-uniform refinement with per-particle defocus refinement. **f,** Angular distribution of E-60 particle images in the final map.

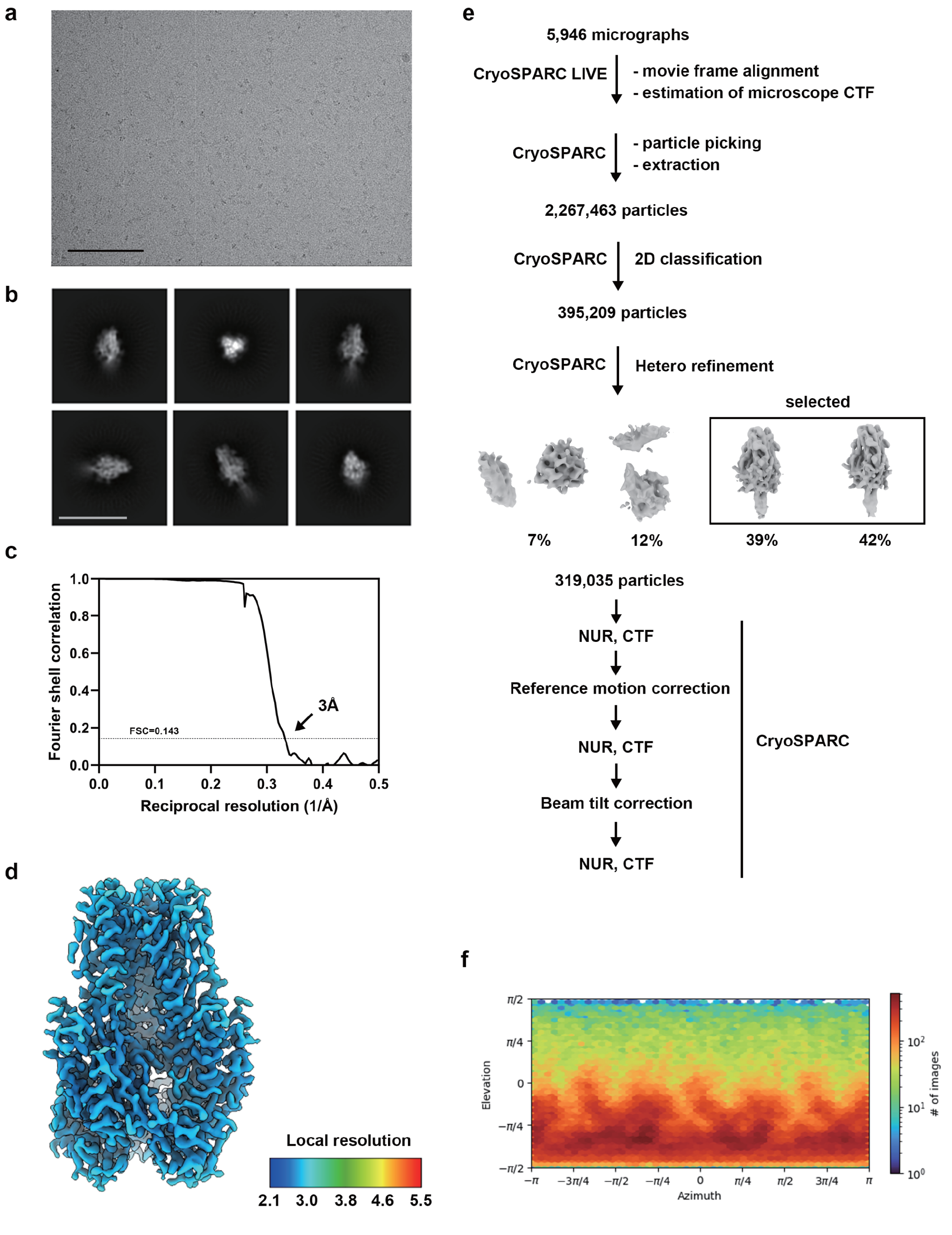

Supplementary Figure 5. CryoEM data collection and refinement of SARS-CoV-2 S_2 E-

69. a-b, Representative electron micrograph (a) and 2D class averages (b) of SARS-CoV-2 S_2 E-69 embedded in vitreous ice. The scale bar represents 100 nm (a) or 200Å (b). **c,** Gold-standard Fourier shell correlation curve for the cryoEM reconstruction. The 0.143 cutoff is indicated with a gray dashed line. **d,** SARS-CoV-2 S_2 E-69 colored by local resolution as determined using cryoSPARC. **e,** Data processing flowchart. NUR, CTF: non-uniform refinement with per-particle defocus refinement. **f,** Angular distribution of E-69 particle images in the final map.

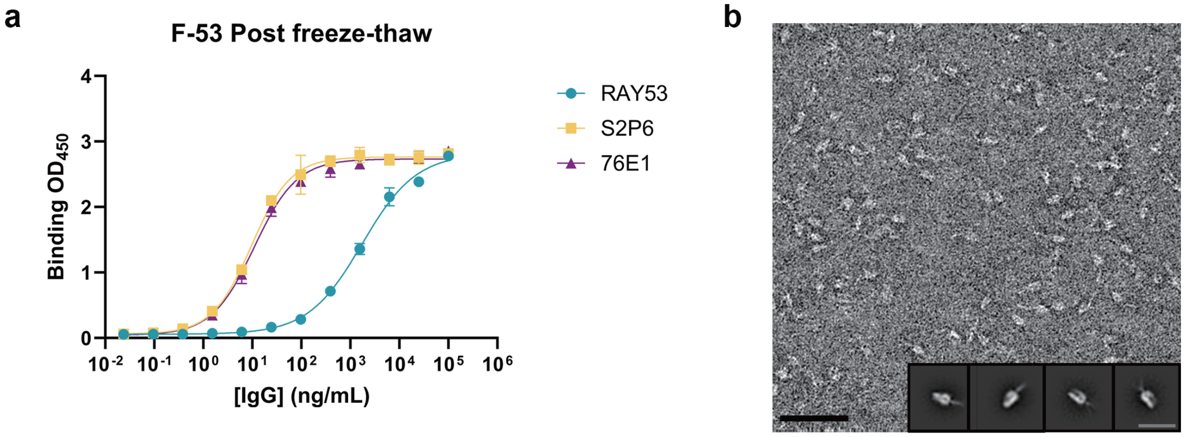

Supplementary Figure 6. Characterization of SARS-CoV-2 S_2 prefusion design F-53. a, Evaluation of binding of a panel of monoclonal antibodies to SARS-CoV-2 S_2 F-53 by ELISA. Each data point represents the mean of three technical replicates and SD are shown with bars. **b,** EM analysis of negatively stained purified F-53. Insets: 2D class averages. The scale bar represents 50 nm (black) or 200 Å (insets, gray).

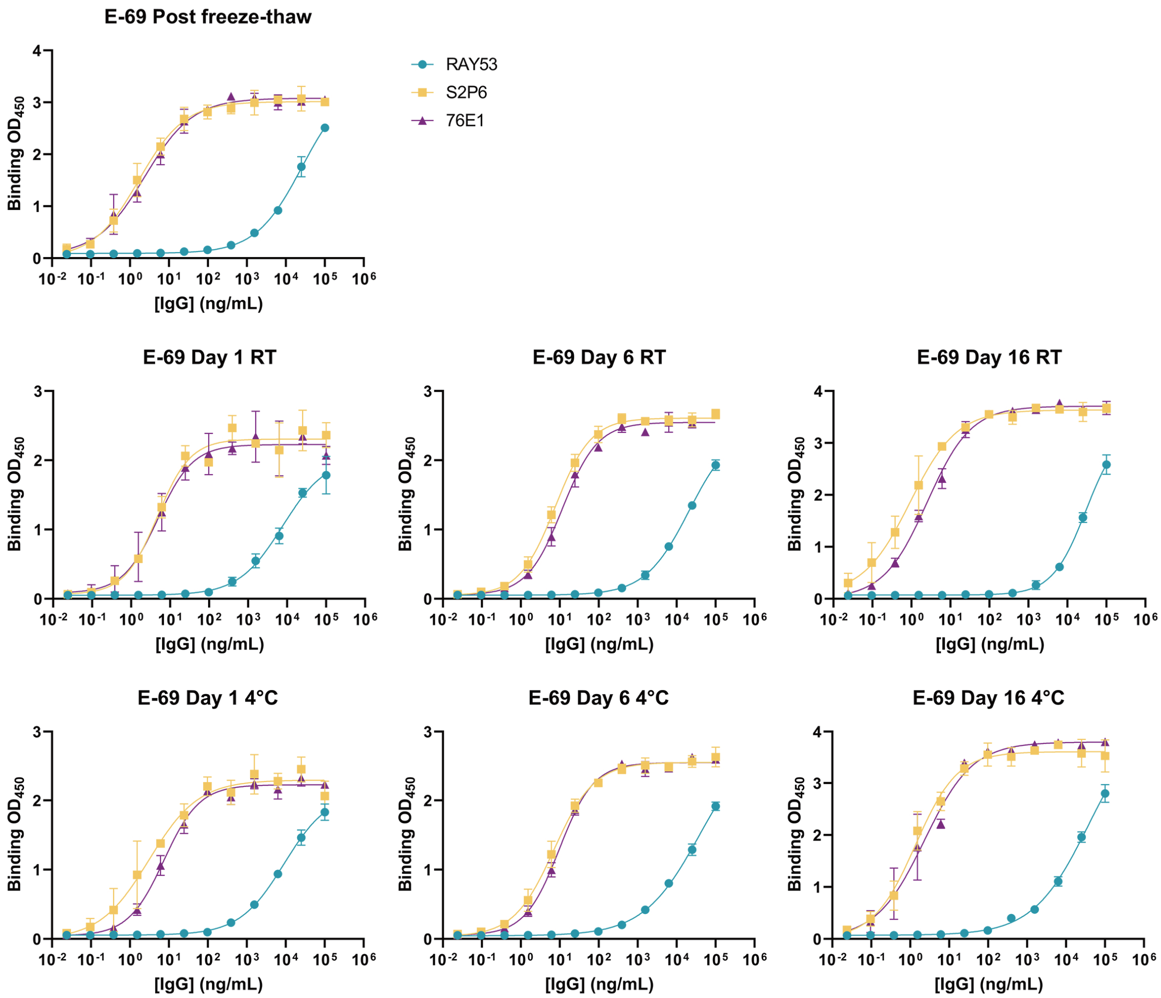

Supplementary Figure 7. Retention of antigenicity of SARS-CoV-2 S_2 E-69. Dose-response curves for evaluation of binding of a panel of monoclonal antibodies to SARS-CoV-2 S_2 E-69 under various storage conditions measured by ELISA. Each data point represents the mean of three technical replicates and SD are shown with bars.

a

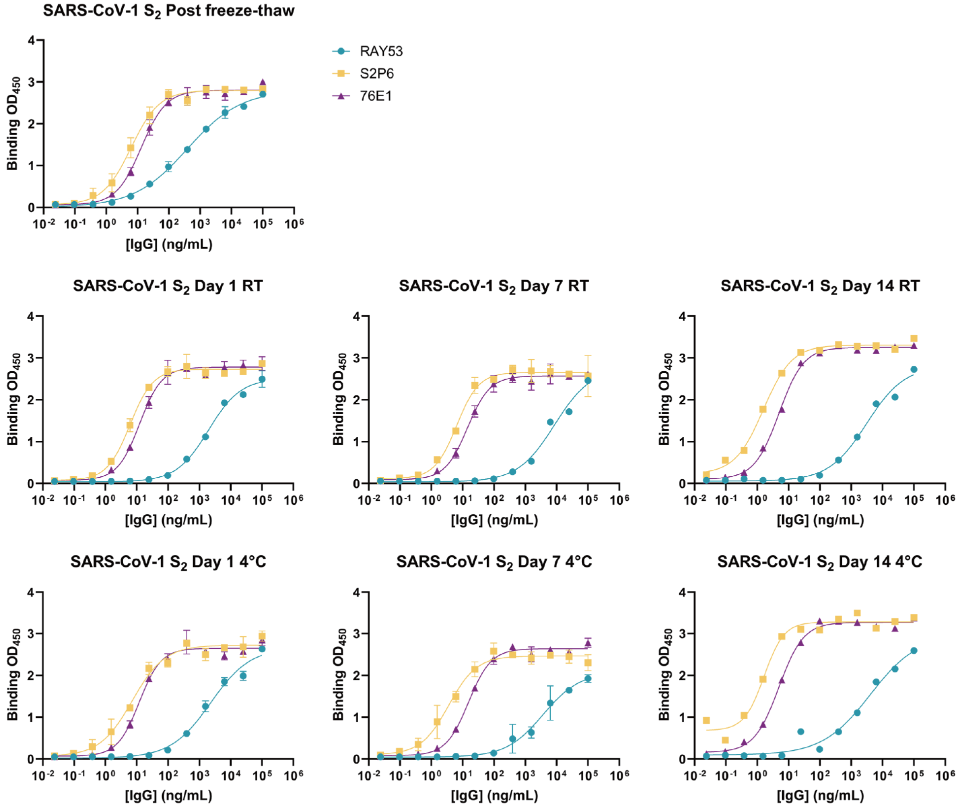
SARS-CoV-1 S2 Post freeze-thaw
RAY53
S2P6
76E1
SARS-CoV-1 S2 Day 1 RT
SARS-CoV-1 S2 Day 7 RT
SARS-CoV-1 S2 Day 14 RT
SARS-CoV-1 S2 Day 1 4°C
SARS-CoV-1 S2 Day 7 4°C
SARS-CoV-1 S2 Day 14 4°C
Binding OD450
[IgG] (ng/mL)
0
1
2
3
4
10-2
10-1
100
101
102
103
104
105
106

b

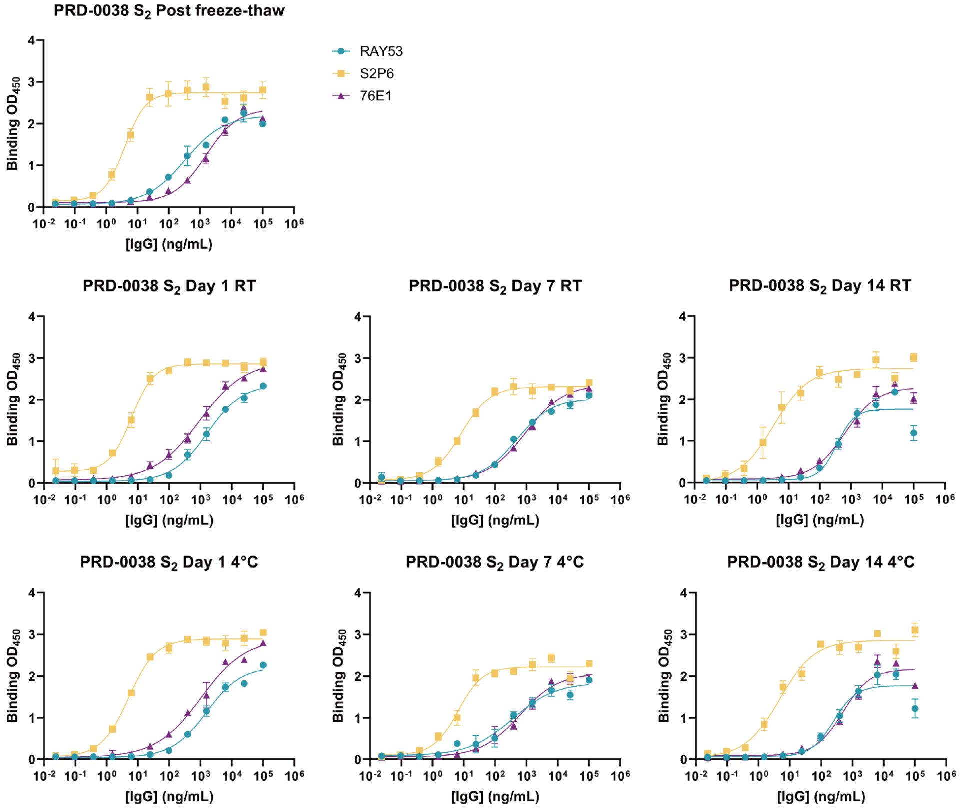
PRD-0038 S2 Post freeze-thaw
RAY53
S2P6
76E1
PRD-0038 S2 Day 1 RT
PRD-0038 S2 Day 7 RT
PRD-0038 S2 Day 14 RT
PRD-0038 S2 Day 1 4°C
PRD-0038 S2 Day 7 4°C
PRD-0038 S2 Day 14 4°C
Binding OD450
[IgG] (ng/mL)
0
1
2
3
4
10-2
10-1
100
101
102
103
104
105
106

Supplementary Figure 8. Retention of antigenicity of SARS-CoV-1 and PRD-0038 prefusion S_2 designed constructs. a-b, Evaluation of binding of a panel of monoclonal antibodies to SARS-CoV-1 S_2 (a) and PRD-0038 S_2 (b) under various storage conditions measured by ELISA. Each data point for SARS-CoV-1 represents the mean of two technical replicates and SD are shown with bars (except for SARS-CoV-1 Day14 RT and 4˚C which comprise a single technical replicate and Post-freeze thaw which comprises three technical replicates). Each data point for PRD-0038 represents the mean of three technical replicates and SD are shown with bars (PRD-0038 Post freeze-thaw which comprises two technical replicates).

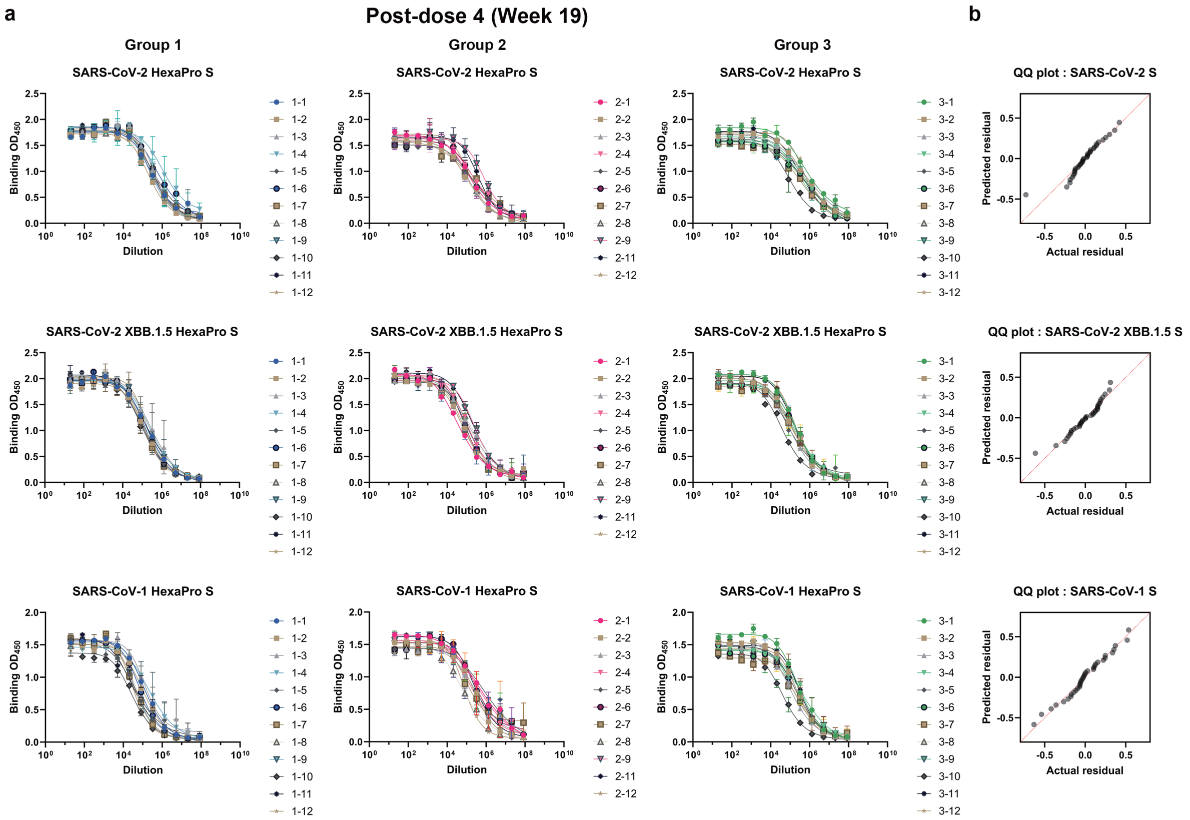

Supplementary Figure 9. Analysis of vaccine-elicited serum antibody binding titers against various S trimers by ELISA. a, Dose-response curves of serum antibody binding to SARS-CoV-2 Hexapro S, XBB.1.5 Hexapro S, and SARS-CoV-1 Hexapro S using sera obtained 2 weeks post dose 4. Each data point represents the mean of two technical replicates and SD are shown with bars. One representative out of two biological replicates is shown (n=12 for groups 1 &3, n=11 for group 2). **b,** QQ plots of residuals for the mean of log(ED_{50}) values from two biological replicates of ELISA.

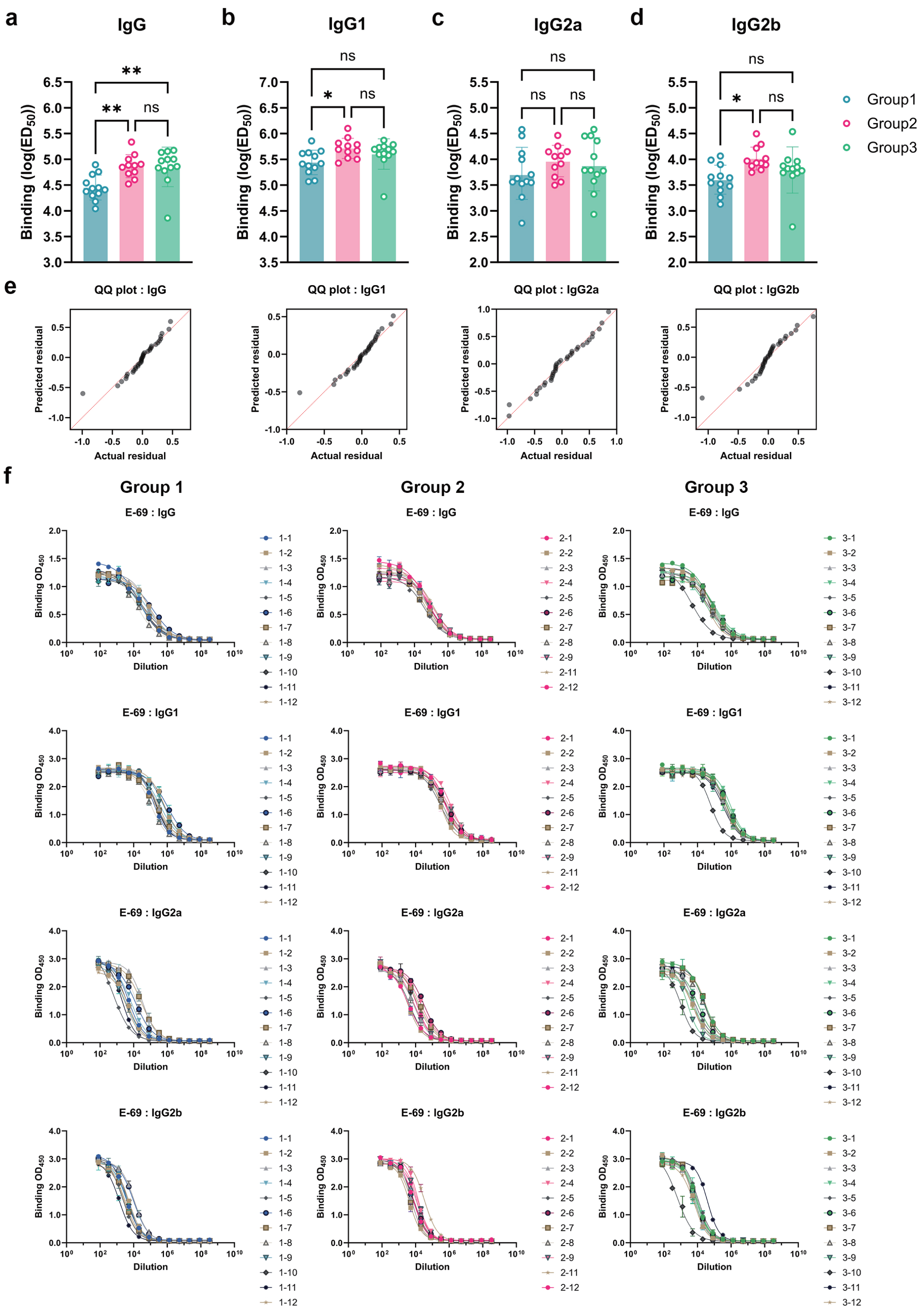

Supplementary Figure 10. Analysis of vaccine-elicited serum binding titers for different IgG subclasses against SARS-CoV-2 E-69. a-d, Analysis of IgG (a), IgG1 (b),

IgG2a (c), and IgG2b (d) binding titers against E-69 analyzed by ELISAs using sera obtained five weeks post dose 4. Geometric means are shown as bars with SD. Each data point represents the mean of two biological replicates each comprising two technical replicates. (n=12 for groups 1 &3, n=11 for group 2). **e,** QQ plots of residuals for the mean of log(ED_{50}) values from two biological replicates of ELISA. **f**, Dose-response curves of serum antibody binding to E-69. Each data point represents the mean of two technical replicates and SD are shown with bars. One representative out of two biological replicates is shown. (n=12 for groups 1 &3, n=11 for group 2). Comparisons between multiple groups for (a-d) were made by ordinary one-way analysis of variance (ANOVA) followed by Tukey's multiple comparisons test. ns : P>0.05, * : P<=0.05, ** : P <=0.01, *** : P<=0.001, **** : P<=0.0001. (a) P=0.0036 and P=0.0044 for the comparisons made between groups1&2 and groups 1&3, respectively. (b) P=0.0277 for the comparison made between groups1&2. (d) P=0.0114 for the comparison made between groups1&2.

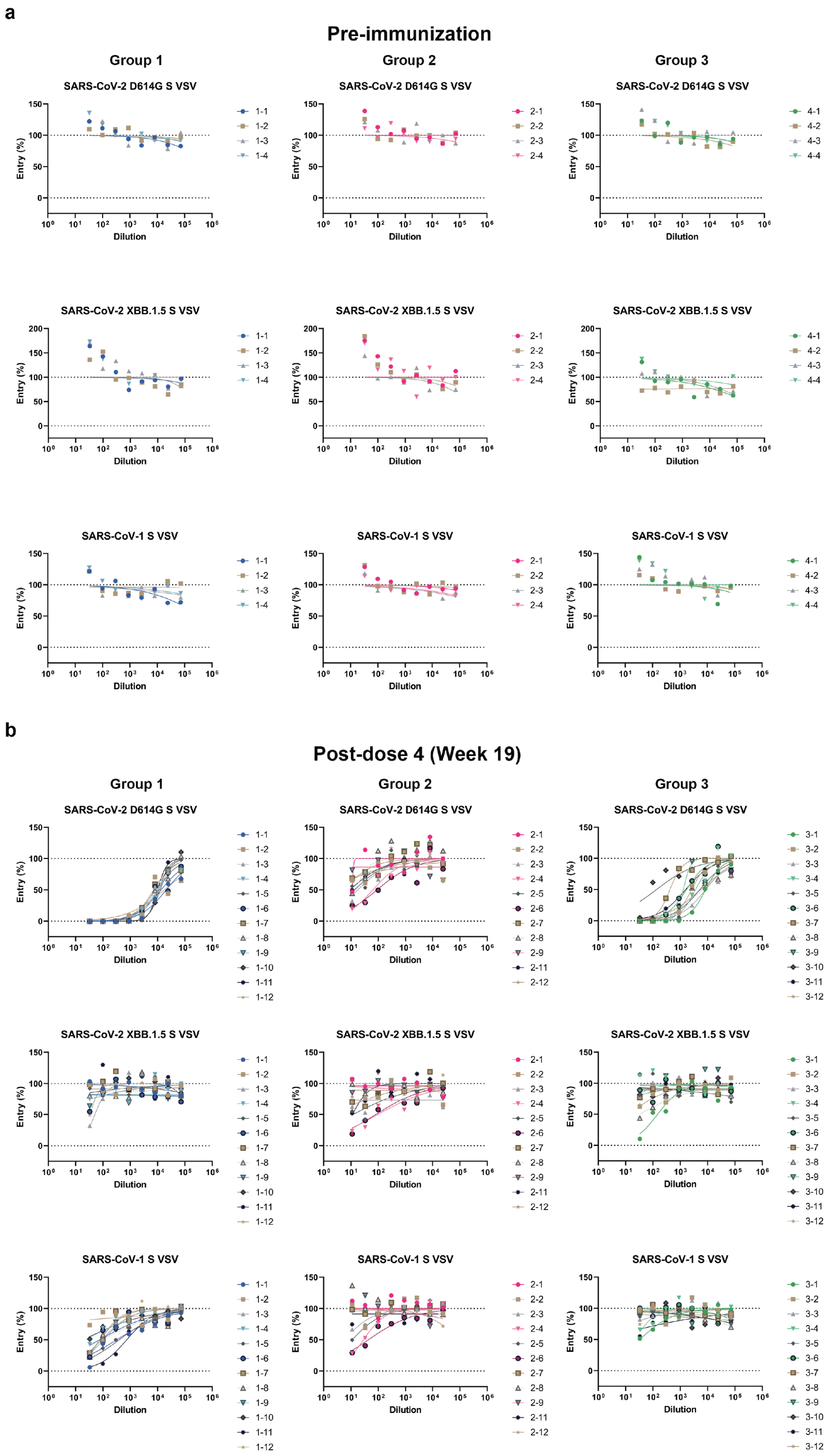
a
Pre-immunization
Group 1
Group 2
Group 3
SARS-CoV-2 D614G S VSV
SARS-CoV-2 XBB.1.5 S VSV
SARS-CoV-1 S VSV
Entry (%)
Dilution
1-1
1-2
1-3
1-4
2-1
2-2
2-3
2-4
4-1
4-2
4-3
4-4
b
Post-dose 4 (Week 19)
Group 1
Group 2
Group 3
SARS-CoV-2 D614G S VSV
SARS-CoV-2 XBB.1.5 S VSV
SARS-CoV-1 S VSV
Entry (%)
Dilution
1-1
1-2
1-3
1-4
1-5
1-6
1-7
1-8
1-9
1-10
1-11
1-12
2-1
2-2
2-3
2-4
2-5
2-6
2-7
2-8
2-9
2-11
2-12
3-1
3-2
3-3
3-4
3-5
3-6
3-7
3-8
3-9
3-10
3-11
3-12

Supplementary Figure 11. Analysis of vaccine-elicited serum neutralizing antibody titers.

a-b, Dose-response curves of serum neutralizing antibody titers against the SARS-CoV-2 Wu/G614, XBB.1.5 and SARS-CoV-1 S VSV pseudotypes using sera obtained prior to immunization (a) and two weeks post dose 4 (b). The color key indicates mouse IDs. Representative curves from one out of three biological replicates are shown.
