# Supplementary material for: A broadly generalizable stabilization strategy for sarbecovirus fusion machinery vaccines
Source: Nat Commun. 2024 Jun 28;15:5496. doi: 10.1038/s41467-024-49656-5 (PMC11214633; doi:10.1038/s41467-024-49656-5)
Supplement: Supplementary file 3 — Reporting Summary [file 41467_2024_49656_MOESM3_ESM.pdf]

nature portfolio

Corresponding author(s): David Veesler

Last updated by author(s): Jun 4, 2024

Reporting Summary

Nature Portfolio wishes to improve the reproducibility of the work that we publish. This form provides structure for consistency and transparency in reporting. For further information on Nature Portfolio policies, see our Editorial Policies and the Editorial Policy Checklist.

Statistics

For all statistical analyses, confirm that the following items are present in the figure legend, table legend, main text, or Methods section.

n/a	Confirmed	
☐	☒	The exact sample size (*n*) for each experimental group/condition, given as a discrete number and unit of measurement
☐	☒	A statement on whether measurements were taken from distinct samples or whether the same sample was measured repeatedly
☐	☒	The statistical test(s) used AND whether they are one- or two-sided<br>*Only common tests should be described solely by name; describe more complex techniques in the Methods section.*
☐	☒	A description of all covariates tested
☐	☐	A description of any assumptions or corrections, such as tests of normality and adjustment for multiple comparisons
☐	☒	A full description of the statistical parameters including central tendency (e.g. means) or other basic estimates (e.g. regression coefficient) AND variation (e.g. standard deviation) or associated estimates of uncertainty (e.g. confidence intervals)
☐	☒	For null hypothesis testing, the test statistic (e.g. *F*, *t*, *r*) with confidence intervals, effect sizes, degrees of freedom and *P* value noted<br>*Give P values as exact values whenever suitable.*
☒	☐	For Bayesian analysis, information on the choice of priors and Markov chain Monte Carlo settings
☒	☐	For hierarchical and complex designs, identification of the appropriate level for tests and full reporting of outcomes
☒	☐	Estimates of effect sizes (e.g. Cohen's *d*, Pearson's *r*), indicating how they were calculated

Our web collection on statistics for biologists contains articles on many of the points above.

Software and code

Policy information about availability of computer code

Data collection	Leginon-v3.5
Data analysis	Coot-v0.9.8.1, CyoSPARC , GraphPad Prism-v10.1.1, Relion-v3.1, Rosetta, UCSF Chimera-v1.16, UCSF ChimeraX-v1.4, WARP-v1.10, AlphaFold2, Phenix-v1.20, Molprobity, Privateer

For manuscripts utilizing custom algorithms or software that are central to the research but not yet described in published literature, software must be made available to editors and reviewers. We strongly encourage code deposition in a community repository (e.g. GitHub). See the Nature Portfolio guidelines for submitting code & software for further information.

Data

Policy information about availability of data

All manuscripts must include a data availability statement. This statement should provide the following information, where applicable:

- Accession codes, unique identifiers, or web links for publicly available datasets
- A description of any restrictions on data availability
- For clinical datasets or third party data, please ensure that the statement adheres to our policy

The sharpened and unsharpened cryoEM reconstructions of the prefusion-stabilized SARS-CoV-2 fusion machinery designs have been deposited in the Electron Microscopy Data Bank (EMDB) under accession codes EMD-43435 [https://www.ebi.ac.uk/pdbe/entry/emdb/EMD-43435] (E-31); EMD-43436 [https://www.ebi.ac.uk/pdbe/entry/emdb/EMD-43436] (E-60); and EMD-43437 [https://www.ebi.ac.uk/pdbe/entry/emdb/EMD-43437] (E-69). The atomic models of

prefusion-stabilized SARS-CoV-2 fusion machinery designs have been deposited in the Protein Data Bank (PDB) under accession codes PDB 8VQ9 [https://doi.org/10.2210/pdb8VQ9/pdb] (E-31); PDB 8VQA [https://doi.org/10.2210/pdb8VQA/pdb] (E-60); and PDB 8VQB [https://doi.org/10.2210/pdb8VQB/pdb] (E-69). Other data will be available from the corresponding author upon request. For the structures that have been referenced in the text are accessible under the PDB accession code PDB 6VXX [https://doi.org/10.2210/pdb6VXX/pdb]; PDB 8DYA [https://doi.org/10.2210/pdb8DYA/pdb]; PDB 6XRB [https://doi.org/10.2210/pdb6XRB/pdb]; PDB 5X58 [https://doi.org/10.2210/pdb5X58/pdb]; and PDB 8U29 [https://doi.org/10.2210/pdb8U29/pdb].
The source data underlying Figures 1c-d, 2c, 3j-m, 4b-g, 5a-d, and Supplementary Figures 6a, 7, 8a-b, 9a, 10a-f, and 11a-b are provided as a Source Data file. Other data will be available from the corresponding author upon request.

Research involving human participants, their data, or biological material

Policy information about studies with human participants or human data. See also policy information about sex, gender (identity/presentation), and sexual orientation and race, ethnicity and racism.

Reporting on sex and gender	*Use the terms sex (biological attribute) and gender (shaped by social and cultural circumstances) carefully in order to avoid confusing both terms. Indicate if findings apply to only one sex or gender; describe whether sex and gender were considered in study design; whether sex and/or gender was determined based on self-reporting or assigned and methods used. Provide in the source data disaggregated sex and gender data, where this information has been collected, and if consent has been obtained for sharing of individual-level data; provide overall numbers in this Reporting Summary. Please state if this information has not been collected. Report sex- and gender-based analyses where performed, justify reasons for lack of sex- and gender-based analysis.*
Reporting on race, ethnicity, or other socially relevant groupings	*Please specify the socially constructed or socially relevant categorization variable(s) used in your manuscript and explain why they were used. Please note that such variables should not be used as proxies for other socially constructed/relevant variables (for example, race or ethnicity should not be used as a proxy for socioeconomic status). Provide clear definitions of the relevant terms used, how they were provided (by the participants/respondents, the researchers, or third parties), and the method(s) used to classify people into the different categories (e.g. self-report, census or administrative data, social media data, etc.) Please provide details about how you controlled for confounding variables in your analyses.*
Population characteristics	*Describe the covariate-relevant population characteristics of the human research participants (e.g. age, genotypic information, past and current diagnosis and treatment categories). If you filled out the behavioural & social sciences study design questions and have nothing to add here, write "See above."*
Recruitment	*Describe how participants were recruited. Outline any potential self-selection bias or other biases that may be present and how these are likely to impact results.*
Ethics oversight	*Identify the organization(s) that approved the study protocol.*

Note that full information on the approval of the study protocol must also be provided in the manuscript.

Field-specific reporting

Please select the one below that is the best fit for your research. If you are not sure, read the appropriate sections before making your selection.

☒ Life sciences ☐ Behavioural & social sciences ☐ Ecological, evolutionary & environmental sciences

For a reference copy of the document with all sections, see nature.com/documents/nr-reporting-summary-flat.pdf

Life sciences study design

All studies must disclose on these points even when the disclosure is negative.

Sample size	Sample size was chosen based on prior experience with the mice model.
Data exclusions	No data were excluded from the analysis.
Replication	Experimental assays were performed at least in two or three independent replicates. Each replicates was performed with 1~3 technical replicates. All replicates were reproducible.
Randomization	Randomization and blinding were not applicable due to pre-defined housing conditions.
Blinding	Analyses were blinded with coded samples.

Reporting for specific materials, systems and methods

We require information from authors about some types of materials, experimental systems and methods used in many studies. Here, indicate whether each material, system or method listed is relevant to your study. If you are not sure if a list item applies to your research, read the appropriate section before selecting a response.

Materials & experimental systems

n/a	Involved in the study	
☐	☒	Antibodies
☐	☒	Eukaryotic cell lines
☒	☐	Palaeontology and archaeology
☐	☒	Animals and other organisms
☒	☐	Clinical data
☒	☐	Dual use research of concern
☒	☐	Plants

Methods

n/a	Involved in the study	
☒	☐	ChIP-seq
☒	☐	Flow cytometry
☒	☐	MRI-based neuroimaging

Antibodies

Antibodies used	Goat anti-Human IgG Fc Secondary Antibody, HRP (Thermo Fisher Scientific - Invitrogen™, Cat#A18817), 76E1, RAY53, S2P6, Goat anti-mouse IgG (H+L) Secondary Antibody HRP (Thermo Fisher Scientific - Invitrogen™, Cat#62-6520), Peroxidase AffiniPure™ Goat Anti-Mouse IgG Fcγ subclass 1 specific (Jackson Immuno Research, 115-035-205), Peroxidase AffiniPure™ Goat Anti-Mouse IgG Fcγ subclass 2a specific (Jackson Immuno Research, 115-035-206), AffiniPure™ Goat Anti-Mouse IgG Fcγ subclass 2b specific (Jackson Immuno Research, 115-035-207)
Validation	We described 76E1, RAY53, and S2P6 monoclonal antibodies in the study all of which are previously reported in indicated studies. Target validation of these antibodies was performed with multiple binding assays. Reactivity of secondary antibodies listed above is based on the information on manufacturer's homepages.

Eukaryotic cell lines

Policy information about cell lines and Sex and Gender in Research

Cell line source(s)	Cell lines used in this study were obtained from HEK293T (ATCC, CRL-11268), Expi293F (Thermo Fisher Scientific, A145277) and VeroE6-TMPRSS2 (JCRB1819).
Authentication	None of the cell lines used were authenticated.
Mycoplasma contamination	Cell lines were not tested for mycoplasma contamination.
Commonly misidentified lines (See ICLAC register)	No commonly misidentified cell lines were used in the study.

Animals and other research organisms

Policy information about studies involving animals; ARRIVE guidelines recommended for reporting animal research, and Sex and Gender in Research

Laboratory animals	Female BALB/c mice were purchased from Envigo (order code 047) at 7 weeks of age.
Wild animals	This study did not involve wild animals.
Reporting on sex	Only female mice were used for reproducibility of the model which was set up with female animals.
Field-collected samples	No field collected samples were used in this study.
Ethics oversight	Animal studies were carried out in an accordance with the Department of Comparative Medicine at the University of Washington, Seattle, accredited by the Association for Assessment and Accreditation of Laboratory Animal Care (AAALAC). Animal experiments were conducted in accordance with the University of Washington’s Institutional Animal Care and Use Committee.

Note that full information on the approval of the study protocol must also be provided in the manuscript.

Plants

Seed stocks	*Report on the source of all seed stocks or other plant material used. If applicable, state the seed stock centre and catalogue number. If plant specimens were collected from the field, describe the collection location, date and sampling procedures.*
Novel plant genotypes	*Describe the methods by which all novel plant genotypes were produced. This includes those generated by transgenic approaches, gene editing, chemical/radiation-based mutagenesis and hybridization. For transgenic lines, describe the transformation method, the number of independent lines analyzed and the generation upon which experiments were performed. For gene-edited lines, describe the editor used, the endogenous sequence targeted for editing, the targeting guide RNA sequence (if applicable) and how the editor was applied.*
Authentication	*Describe any authentication procedures for each seed stock used or novel genotype generated. Describe any experiments used to assess the effect of a mutation and, where applicable, how potential secondary effects (e.g. second site T-DNA insertions, mosiacism, off-target gene editing) were examined.*
